# Characterization of NAD^+^/NADP^+^-Specific Isocitrate Dehydrogenases From Oleaginous Fungus *Mortierella alpina* Involved in Lipid Accumulation

**DOI:** 10.3389/fnut.2021.746342

**Published:** 2021-10-21

**Authors:** Xin Tang, Xiaoqi Sun, Xuxu Wang, Hao Zhang, Yong Q. Chen, Jianxin Zhao, Haiqin Chen, Wei Chen

**Affiliations:** ^1^State Key Laboratory of Food Science and Technology, Jiangnan University, Wuxi, China; ^2^School of Food Science and Technology, Jiangnan University, Wuxi, China; ^3^National Engineering Research Center for Functional Food, Jiangnan University, Wuxi, China; ^4^Wuxi Translational Medicine Research Center and Jiangsu Translational Medicine Research Institute Wuxi Branch, Wuxi, China; ^5^Beijing Innovation Centre of Food Nutrition and Human Health, Beijing Technology and Business University (BTBU), Beijing, China

**Keywords:** *Mortierella alpina*, isocitrate dehydrogenase, homologous overexpression, lipid accumulation, mechanism

## Abstract

*Mortierella alpina* has a strong capacity for lipid accumulation. Isocitrate dehydrogenase (IDH) plays an important role in affecting the flow of intracellular carbon sources and reducing power NADPH for lipid biosynthesis. In this study, the effect of various IDHs (NAD^+^- and NADP^+^-specific) in *M. alpina* on the lipid accumulation was investigated through homologous overexpression. The results showed that the transcription level and enzyme activity of the IDHs from *M. alpina* (MaIDHs) in homologous overexpressing strains were higher than those of the control strain, but that their biomass was not significantly different. Among the various NAD^+^-specific MaIDH1/2/3 overexpression, NAD^+^-MaIDH3 reduced total lipid content by 12.5%, whereas overexpression NAD^+^-MaIDH1 and NAD^+^-MaIDH2 had no effect on fatty acid content. Intracellular metabolites analysis indicated that the overexpression NAD^+^-MaIDH3 strain had reduced the fatty acid accumulation, due to its greater carbon flux with the tricarboxylic acid cycle and less carbon flux with fatty acid biosynthesis. For the NADP^+^-MaIDH4/5/6 recombinant strains overexpressing only NADP^+^-MaIDH4 enhanced the total fatty acid content by 8.2%. NADPH analysis suggested that this increase in lipid accumulation may have been due to the great reducing power NADPH is produced in this recombinant strain. This study provides theoretical basis and guidance for the analysis of the mechanism of IDH function and the potential to improve lipid production in *M. alpina*.

## Introduction

*Mortierella alpina* has strong fatty acid synthesis ability and can synthesize a variety of biologically active polyunsaturated fatty acids (PUFAs), including arachidonic acid (AA) and eicosapentaenoic acid (EPA) (1). *Mortierella alpina* is an industrialized strain that produces AA (2), which is an essential nutrient for early human development. At present, AA synthesized by *M. alpina* has become an important additive for infant milk powder (3–5).

Isocitrate dehydrogenase (IDH) is an enzyme that plays an important role in energy metabolism, amino acid synthesis and vitamin synthesis. It catalyzes the oxidative dehydrogenation of isocitrate to form the intermediate product, oxalosuccinic acid, followed by oxidative decarboxylation to produce 2-oxoglutarate (6). IDH has been found in almost all organisms, including animals (7), plants (8), fungi (9), and bacteria (10). IDH can be divided into NAD^+^-specific isocitrate dehydrogenase (NAD^+^-IDH, EC 1.1.1.41) which has NAD^+^ as an electron acceptor and NADP^+^-specific isocitrate dehydrogenase (NADP^+^-IDH, EC 1.1.1.42) which has NADP^+^ as an electron acceptor. NAD^+^-IDH is generally localized in the mitochondrial matrix and is the rate-limiting enzyme of the tricarboxylic acid (TCA) cycle, which is an important metabolic pathway affecting fatty acid synthesis (11). NADP^+^-IDH is mainly localized in eukaryotic chloroplasts (12), mitochondria (13), peroxisomes (14), cytoplasm (15), and prokaryotic cells (16), and serves as one of the sources of NADPH. It plays a crucial role in cell defense against oxidative damage (17–19). Many enzymes are cofactor dependent and they frequently require NADH or NADPH in stoichiometric quantities for their catalytic function (20–22).

Previous studies had shown that isocitrate dehydrogenase is related to lipid accumulation in microorganisms (23–25). When the nitrogen source in culture medium is depleted, the activity of intracellular adenosine monophosphate (AMP) deaminase (AMPD) increases rapidly, catalyzing the conversion of AMP to inosine monophosphate (IMP), and releasing ${\text{NH}}_{4}^{+}$ to supplement the nitrogen source. The activity of NAD^+^-IDH depends on the concentration of intracellular AMP (26, 27); decrease in AMP concentration leads to decrease in the activity of NAD^+^-IDH, which in turn inhibits the tricarboxylic acid cycle. Subsequently, the proliferation rate of oleaginous microbial cells reduces and they start entering the lipid accumulation phase. Under nitrogen-limiting conditions, the decrease in NAD^+^-IDH activity in mitochondria reduces the conversion rate of isocitrate to α-ketoglutaric acid, and isocitrate accumulates in mitochondria producing citric acid via aconitate hydratase. The citrate in the mitochondria is then transported to the cytoplasm via the citrate transporter where it is decomposed by the ATP citrate lyase (ACL) to form oxaloacetate and acetyl-CoA (25, 28). Acetyl-CoA is used as a substrate for lipid biosynthesis in oleaginous microorganisms. In the cytoplasm, NADP^+^-IDH catalyzes a reaction similar to that of NAD^+^-IDH in the mitochondria, and mediates oxidative decarboxylation of isocitrate to form α-ketoglutaric acid. However, unlike NAD^+^-IDH, which produces NADH, NADP^+^-IDH can reduce NADP^+^ in the cytoplasm to produce reducing power NADPH, which together with the malic enzyme and pentose phosphate pathways for fatty acid synthase in lipid-producing microorganisms.

In this study, various isozymes of IDH from *M. alpina* (MaIDH) were investigated for their effect on lipid biosynthesis. This is conducive to improving our understanding the mechanism of lipid accumulation in *M. alpina*, and provides theoretical support for concepts to follow-up on and further improve the efficiency of lipid production in *M. alpina*. Moreover, the research results could be applied to other lipid-producing microorganisms, enhancing its scientific significance and application value.

## Materials and Methods

### Strain, Media, and Culture Conditions

*Mortierella alpina* ATCC 32222 was purchased from the American Type Culture Collection, and the uracil auxotrophic strain CCFM 501 was constructed by our laboratory and used as a recipient strain for transformation (29). The uracil-recovering strain CCFM 505 was used as the control strain for the experiments. *Escherichia coli* Top 10 was used to preserve the constructed recombinant plasmid. *Agrobacterium tumefaciens* CCFM 834 was used for the mediated transformation of *M. alpina* and the plasmid pBIG2-ura5s-ITs contained kanamycin resistance and uracil screening markers was used as an expression vector for genetic manipulation. The *E. coli* Top 10 strain for plasmid storage and construction was cultivated at 37°C in lysogeny broth (LB) medium (10 g/L tryptone, 5 g/L yeast extract, and 10 g/L NaCl). *A. tumefaciens* CCFM 834 was grown on yeast extract-peptone (YEP) medium (10 g/L tryptone, 10 g/L yeast extract, and 5 g/L NaCl) at 28°C. The uracil-auxotrophic *M. alpina* strain CCFM 501 and wild-type *M. alpina* ATCC 32222 were maintained on glucose-yeast extract (GY) solid medium (30 g/L glucose, 5 g/L yeast extract, 2 g/L KNO_3_, 1 g/L NaH_2_PO_4_, 0.3 g/L MgSO_4_·7H_2_O, and 15 g/L agar), supplemented with 0.1 g/L uracil when necessary. For flask culture, *M. alpina* spores were inoculated into the broth medium (20 g/L glucose, 5 g/L yeast extract, 10 g/L KNO_3_, 1 g/L KH_2_PO_4_, and 0.25 g/L MgSO_4_·7H_2_O) at 28°C for 48 h and transferred into a new broth medium (1%, v/v). This was repeated for three generations, and mycelium cultured in the 100 mL modified Kendrick medium (50 g/L glucose, 2.0 g/L diammonium tartrate, 7.0 g/L KH_2_PO_4_, 2.0 g/L Na_2_HPO_4_, 1.5 g/L MgSO_4_·7H_2_O, 1.5 g/L yeast extract, 0.1 g/L CaCl_2_·2H_2_O, 8 mg/L FeCl_3_·6H_2_O, 1 mg/L ZnSO_4_·7H_2_O, 0.1 mg/L CuSO_4_·5H_2_O, 0.1 mg/L Co(NO_3_)_2_·6H_2_O, and 0.1 mg/L MnSO_4_·5H_2_O) held in 250 mL flasks for 7 days at 28°C, with agitation at a shaft speed of 200 rpm.

### Construction of the Recombinant Strain

Specific primers were designed based on the *M. alpina* IDH nucleic acid sequence; the primers are listed in Supplementary Table S1. *Mortierella alpina* cDNA was used as a template, and specific amplification of the target gene was used for PCR amplification to obtain the target fragment. The PCR amplification reaction was carried out using a KOD high-fidelity enzyme system as per manufacturer's instructions. After the PCR amplification product was verified by nucleic acid electrophoresis, the PCR product was subjected to gelation recovery and purification. Meanwhile, *E. coli* Top 10 contained the plasmid pBIG2-ura5s-ITs was cultured overnight in an LB liquid medium contained Kanamycin, and the plasmid was extracted using plasmid extraction kit as per manufacturer's instructions. The purified PCR product and the extracted plasmid were digested with the same restriction endonucleases, according to specified reaction conditions for each enzyme, subsequently, the digested products were purified using a gel recovery purification kit and the ligation reaction was prepared according to the specifications of T4 ligase to enable the ligation of the target gene to the expression vector pBIG2-ura5-ITs. *E. coli* Top 10 was used to prepare competent cells, and 5 μL of the ligation product was added to the competent cells for electroporation. In this experiment, the target gene fragment on the original plasmid pBIG2-ura5s-ITs was replaced with the recombinant gene fragment, to construct a recombinant binary expression vector. The recombinant plasmid was named pBIG2-ura5s-MaIDH1/2/3/4/5/6 and transformed into *E. coli* Top 10 for preservation.

### *A. tumefaciens*-Mediated Transformation (ATMT)

ATMT was performed as previously described (1, 29). *A. tumefaciens* stored at −80°C were activated on the YEP screening plates. After picking up single colonies, the cells were inoculated in YEP liquid medium contained Kanamycin and Rifampicin, and cultured at 28°C and 200 rpm for 36–48 h in the dark, until the medium became bright orange. The minimum medium (MM) was inoculated at 1% bacterial solution and cultured. When the OD_600_ value of the bacterial solution in the MM medium reached 1.2–1.5, it was inoculated into 20 mL of inducing medium (IM) supplemented with acetosyringone. The initial OD_600_ was adjusted between 0.15 and 0.20, and incubated at 28°C and 200 rpm away light until the OD_600_ was ~0.8. The uracil-deficient spores of *M. alpina* CCFM 501 were collected in a sterile 50 mL centrifuge tube with broth liquid medium, and incubated at 28°C for 24 h. The *A. tumefaciens* liquid and *M. alpina* spore solution were mixed and applied to IM solid medium coated with cellophane, and cultured at 28°C for 48 h in the dark. After a layer of hyphae grew on the surface of the IM, the membrane on the IM was transferred to the uracil-free synthetic-complete medium with cefotaxime and spectinomycin (SC-CS), and the culture was allowed to incubate at 28°C. After 3 days, the new colonies grew. The newly grown colonies were picked and cultured on fresh SC-CS medium for three consecutive cycles, and the transformants were stably cultured on the SC-CS plates. The compositions of IM, MM, and SC-CS were described previously (30, 31). The recombinant binary expression vector was transformed into uracil auxotrophic *M. alpina* by *A. tumefaciens* CCFM 834 to complete the development of the recombinant strain. The recombinant strains were named *M. alpina-MaIDH1/2/3/4/5/6*.

### Preparation of Genomic DNA

Suspected positive transformants were picked and inoculated in broth liquid medium for further culturing. The cells were collected, and genomic DNA was extracted using the Biospin Fungal Genomic DNA Extraction Kit (BioFlux Cat #BSC14M1).

### Bioinformatic Analysis of MaIDH

A BLAST local library was constructed using *M. alpina* genome-wide data (32), and the MaIDH sequence was screened and further sequenced with the isocitrate dehydrogenase submitted to NCBI; the functional domain search was performed using NCBI BLASTp analysis. Transcription-level analysis of MaIDH was performed according to transcriptomic data for *M. alpina* ATCC 32222, which was initially determined by the laboratory (33). Subcellular localization prediction analysis and signal peptide analysis of MaIDH in *M. alpina* were performed using the online analysis website TargetP1.1 Server (http://www.cbs.dtu.dk/services/TargetP/). The transmembrane structure prediction analysis of MaIDH in *M. alpina* was carried out using the online analysis website TMHMM Server v. 2.0, (http: //www. cbs. dtu. dk/services/TMHMM/). Throughout the above description of bioinformatics data, the function of each MaIDH isozyme in *M. alpina* was predicted and further investigated.

### Transcript-Level Analysis

Total RNA was extracted using the Trizol lysis method, and 1 μg of total RNA was used as a template to obtain cDNA by reverse transcription according to the instructions of PrimeScript™ RT reagent Kit with gDNA Eraser (Takara Reverse Transcription Kit, RR047A). The cDNA obtained was quantified and stored at −80°C until further use. The cDNA obtained by reverse transcription was used as a template, and the reaction system was prepared in accordance with the instructions of SYBR Green SuperMix. Primer pairs used for quantitative reverse transcription PCR (RT-qPCR) are listed in Supplementary Table S2. RT-qPCR was carried out using the Bio-Rad CFX Connect™ system, to determine the level of expression of the target gene. The 18S rDNA of *M. alpina* was used as the internal reference gene, and the cDNA from prototrophic strain CCFM 505 was used as the control. The relative transcription levels of the target genes were analyzed using the 2^−ΔΔCt^ method.

### Determination of Biomass and Fatty Acid Content of the MaIDH Homologous Overexpressing Strain

The cultured *M. alpina* were collected, the wet cells were washed three times with deionized water, and the excess water that remained on the cells was absorbed using a sheet of filter paper. The cells were placed in a vacuum freeze dryer and lyophilized, and the dry weight of the cells was obtained for calculation of the biomass of the recombinant strain. The weighed cells were ground to a powder in a mortar, and fatty acids were extracted by the methanol chloroform method using pentadecanoic acid (C15:0) as an internal standard. Derivatization was performed using methanolic hydrochloric acid. The extracted fatty acid methyl ester was detected using gas chromatography–mass spectrometry (GC-MS) (32).

### Determination of Enzyme Activity of the Recombinant MaIDH Strain

Extraction of the crude enzyme solution was performed as described by Wynn et al. (34). The mycelia of the cultured *M. alpina* recombinant strain were collected and washed three times with deionized water. Subsequently, the mycelium was obtained by suction filtration using a Buchner funnel, was quickly frozen in liquid nitrogen, placed in a mortar with liquid nitrogen, and ground to a powder form. The powder was placed in a centrifuge tube, and enzyme extraction buffer was added. The tube was centrifuged at 10,000 × g for 10 min at 4°C, and the supernatant was aspirated into a new centrifuge tube. The above procedure was repeated twice, and the supernatant was obtained as a crude enzyme solution. A standard curve was prepared using bovine serum albumin as a standard, and the protein concentration of the crude enzyme solution sample was determined using the BCA method. The enzyme activity assay was performed at 30°C according to the method of Wynn et al. (34). The standard reaction system was maintained at 82.2 mmol/L Tris-HCl, pH 8.0, 1.2 mmol/L NAD^+^ or 0.6 mmol/L NADP^+^, 50 mmol/L DL-isocitrate, and 3 mmol/L MgCl_2_. The catalytic activity of IDH was monitored by the generation of NADH or NADPH at 340 nm.

### Extraction and Analysis of Metabolites of the MaIDH-Homologous Overexpressing Strain

The mycelia of the cultured *M. alpina* recombinant strain were collected and washed three times with physiological saline. Then, the mycelium was obtained by suction filtration using a Buchner funnel, and the mycelium was quenched in liquid nitrogen for 10 s, placed in a mortar that was pre-cooled in advance, and ground to a powder form by adding liquid nitrogen. Approximately 50 mg of ground powder was weighed into a 1.5 mL centrifuge tube and 0.6 mL of aqueous methanol (1:1, v/v) was added. Precisely, 50 μL of 2 mg/mL heneicosanoic acid (C21:0) internal standard was added and left to stand for 30 min at −80°C, then centrifuged at 12,000 × g for 15 min. The supernatant was collected and transferred to a new pre-cooled 1.5 mL centrifuge tube. 0.6 mL aqueous methanol solution (1:1, v/v) was added to the precipitate, the supernatant was extracted twice, and the two supernatants were combined. The collected supernatant was concentrated and dried in vacuum for 4–6 h, and 100 μL of pyridine contained 10 mg/mL methoxyamine hydrochloride was added, followed by a metal bath at 37°C for 90 min. Derivatization was continued by adding 40 μL of MSTFA + 1% TMS and a metal bath at 37°C for 30 min. The derivatized product was analyzed using GC-MS.

### Statistical Analysis

The mean values and the standard errors were calculated from three biological replicates. A statistical analysis of the data was performed by SPSS 24.0 for Windows. One-way analysis of variance (ANOVA) with Duncan's test was conducted on the data, and *p* < 0.05 was considered to indicate that the data were significantly different.

## Results and Discussion

### Bioinformatic Analysis of MaIDH

According to the genome-wide information of *M. alpina* ATCC 32222 (32), six nucleic acid sequences encoding IDH were identified in the genome of *M. alpina*, and named MaIDH1/2/3/4/5/6. Among them, MaIDH1/2/3 are NAD^+^-specific IDHs, and MaIDH4/5/6 are NADP^+^-specific IDHs. IDH is a class of basal metabolic enzymes widely found in bacteria, fungi, animals, plants, and other organisms. To further investigate the functional structure of MaIDH, their conserved domain was analyzed, revealing that all MaIDH belong to the isocitrate dehydrogenase superfamily. Among them, MaIDH1/2/3 were annotated as mitochondrial NAD^+^-specific IDH, suggesting that its subcellular localization may occur in mitochondria. The mitochondrial NAD^+^-specific IDH of eukaryotes functions in a similar manner to NADP^+^-specific IDH and 3-isopropylmalate dehydrogenase (NAD^+^-specific enzymes) in prokaryotes. It is closely tied to basic metabolism, energy production, and amino acid transport in the cell, and is usually an α(2)-β-γ heterotetramer. MaIDH4/5/6 were annotated as eukaryotic NADP-specific IDHs, which may be present in cytoplasm, mitochondria, and chloroplasts, but their amino terminus was very similar to that of the cytoplasmic form, indicating that they may be located in the cytoplasm.

Predictive analysis of the subcellular localization of MaIDH was performed according to the TargetP1.1 Server online analysis website. Mitochondrial targeting peptide results indicate that NAD^+^-specific MaIDH1/2/3 may be highly likely to be localized in mitochondria, and NADP^+^-specific MaIDH4/5/6 is highly likely to be localized in the cytoplasm due to the absence of specific signal peptides. In addition, the transmembrane structure of MaIDH protein was predicted based on online analysis at TMHMM Server v.2.0. The results showed that there was no transmembrane structure in MaIDH1/2/3/4/5/6 indicating that MaIDH is not a transmembrane protein localized on the biofilm. Specific information is listed in Table 1.

**Table 1:** Information for the isocitrate dehydrogenases from *M. alpina* (MaIDHs).

**Gene name**	***M. alpina*** **genome number**	**Length (bp)**	**Coenzyme dependence**	**Subcellular localization prediction**
*MaIDH1*	MA-00067-296	1086	NAD^+^	Mitochondria
*MaIDH2*	MA-00073-258	1077	NAD^+^	Mitochondria
*MaIDH3*	MA-00153-313	1137	NAD^+^	Mitochondria
*MaIDH4*	MA-00090-236	1233	NADP^+^	Cytoplasm
*MaIDH5*	MA-00184-263	1248	NADP^+^	Cytoplasm
*MaIDH6*	MA-00297-491	1191	NADP^+^	Cytoplasm

**Figure 1:**
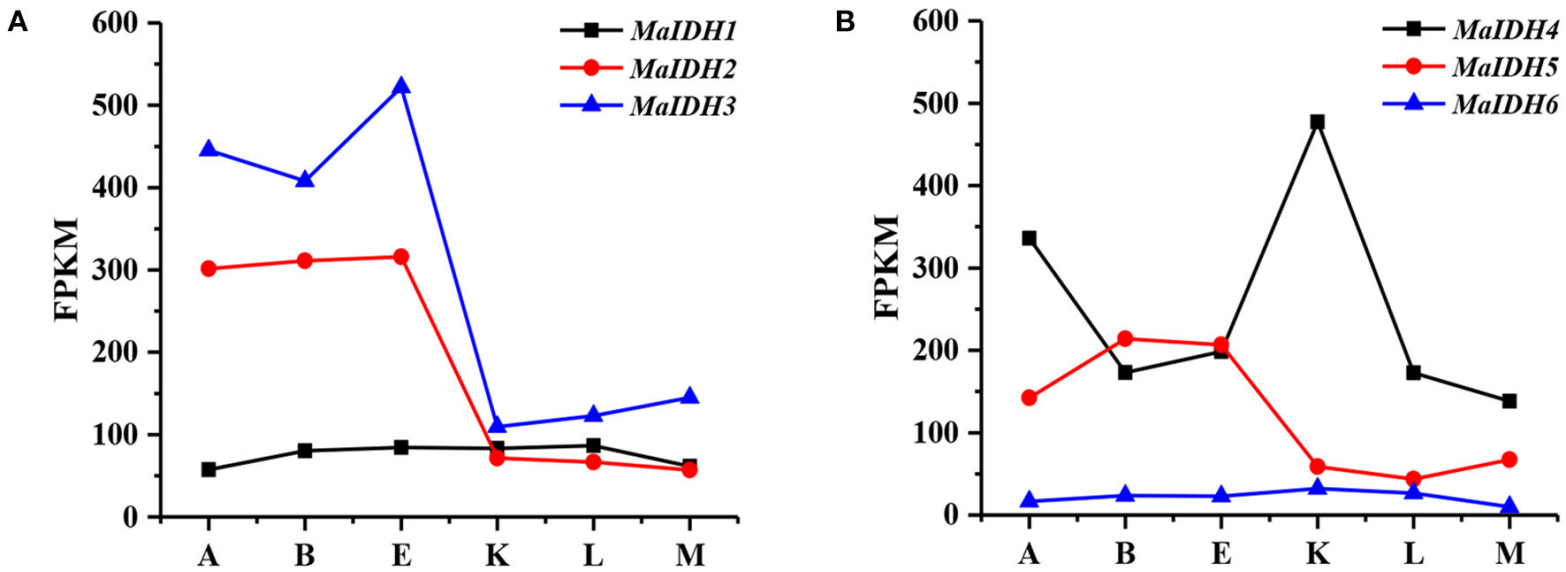
Transcriptome level analysis of isocitrate dehydrogenases from *M. alpina* (MaIDHs) at different time points during the fermentation process. A–K indicate various time points prior to and after nitrogen exhaustion, A: −12 h, B: −2 h, E: −30 min, K: +1 h, L: +12 h, and M: +48 h. **(A)** FPKM of NAD^+^-MaIDHs, **(B)** FPKM of NADP^+^-MaIDHs.

Genes with expression levels that change before and after nitrogen depletion may be key regulators in fatty acid synthesis, and their changes in transcription levels may be responsible for the transformation of *M. alpina* from cell proliferation to lipid accumulation. The transcription level of each MaIDH isozyme was analyzed based on the transcriptome data for *M. alpina*, which was determined in our previous study (33). The time point E, where the nitrogen source was exhausted, was used as a control. As shown in Figure 1, the transcriptional levels of MaIDH genes at different time points during the fermentation process varied. MaIDH1 and MaIDH6 did not change significantly during the entire fermentation process. The FPKM value of MaIDH1 was maintained between 50 and 100, while that of MaIDH6 was always below 35. Compared to the other four MaIDHs, their transcription levels were lower, and were not regulated by the levels of the nitrogen source during fermentation. The transcription levels of MaIDH2 and MaIDH3 after nitrogen depletion were ~5 times lower than before nitrogen depletion (point E to point K), but the FPKM value of *MaIDH3* was always higher than that of MaIDH2. MaIDH5 was downregulated 3.5-fold, and MaIDH4 was the only MaIDH that was up-regulated after nitrogen-restricted; its transcription level was up-regulated 2.5-fold compared to point E.

### Generation of MaIDH-Overexpressing Strains of *M. alpina* by Genetic Engineering

**Figure 2:**
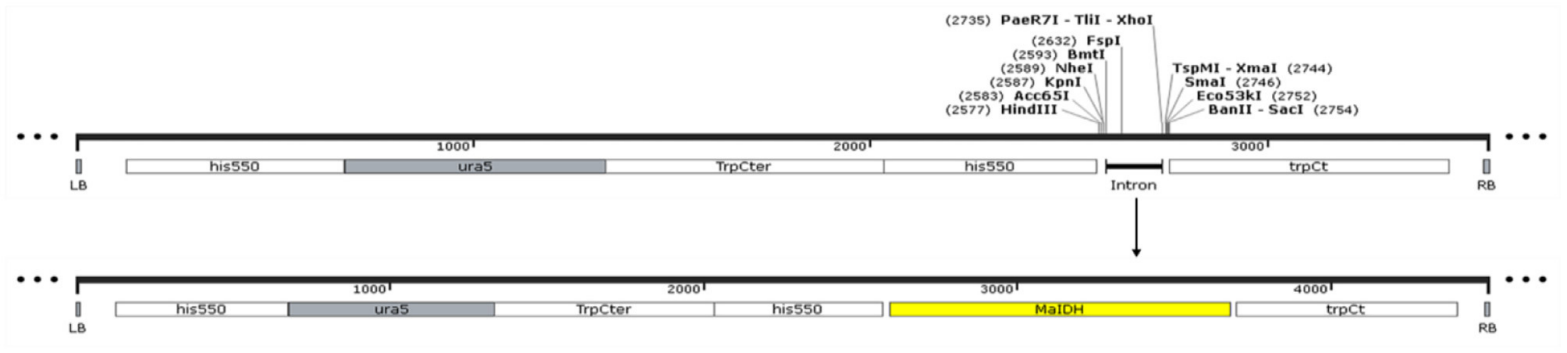
Construction of the vector overexpressing isocitrate dehydrogenases from *M. alpina* (MaIDHs).

**Figure 3:**
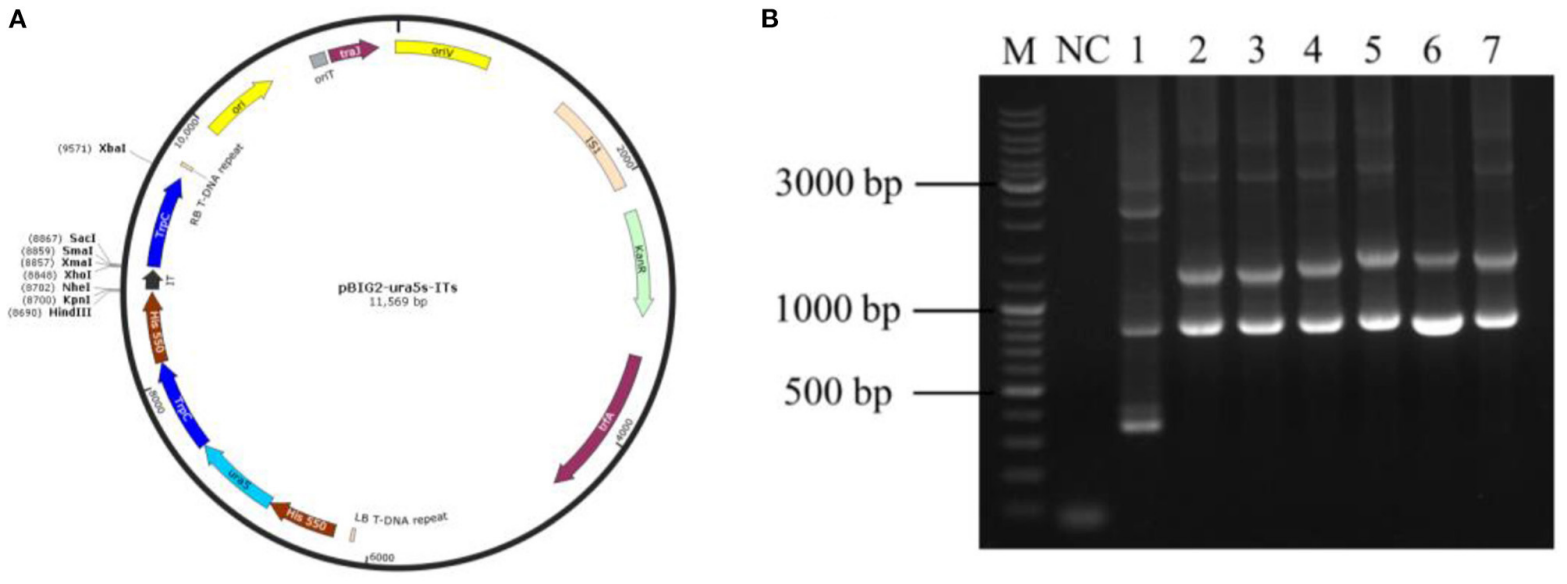
Polymerase chain reaction (PCR) verification of the vector overexpressing isocitrate dehydrogenases from *M. alpina* (MaIDHs). **(A)** pBIG2-ura5s-ITs plasmid map, **(B)** *E. coli* colony PCR assay, M, Marker; NC, Negative control; 1, Empty vector; 2~7, *MaIDH1/2/3/4/5/6* overexpression vectors.

Using the *M. alpina* cDNA as a template, the target gene was subjected to PCR amplification using specific primers for MaIDH1/2/3/4/5/6. The obtained target fragment of MaIDH1/2/3/4/5/6 was ligated to the binary expression vector pBIG2-ura5s-ITs, and the construction process of each recombinant vector of MaIDH is shown in Figure 2. Successfully constructed recombinant binary expression vectors were transformed into *E. coli* Top 10, and positive transformants were selected on the screening plates and verified using the universal primer Hispro F1/TrpCR 1 of pBIG2-ura5s-ITs. The universal primer binds to the same promoter (his550) and terminator (trpCT) in the T-DNA region of the binary expression vector. Thus, two fragments of the uracil nutritional complement sequence ura5 (818 bp) and the target gene (MaIDH) were amplified. The results in Figure 3 showed that the nucleic acid electrophoresis gel pattern showed bright and clear bands at 818 bp and the position of the target gene, which was consistent with the theoretical value. The recombinant binary expression vector of MaIDH was successfully constructed and transferred into *E. coli* Top 10 for preservation. The constructed binary expression vectors were named pBIG2-ura5s-MaIDH1/2/3/4/5/6.

*Agrobacterium tumefaciens*-mediated transformation experiments of *M. alpina* were carried out, and six strains of MaIDH1/2/3/4/5/6 homologous overexpression strains were successfully constructed. Each transformant grew normally after three serial cycles on SC-CS screening medium, indicating that the characteristic traits of the transformants were genetically stable.

### Expression Levels and Enzyme Activity of MaIDH in the Overexpressing Strain

**Figure 4:**
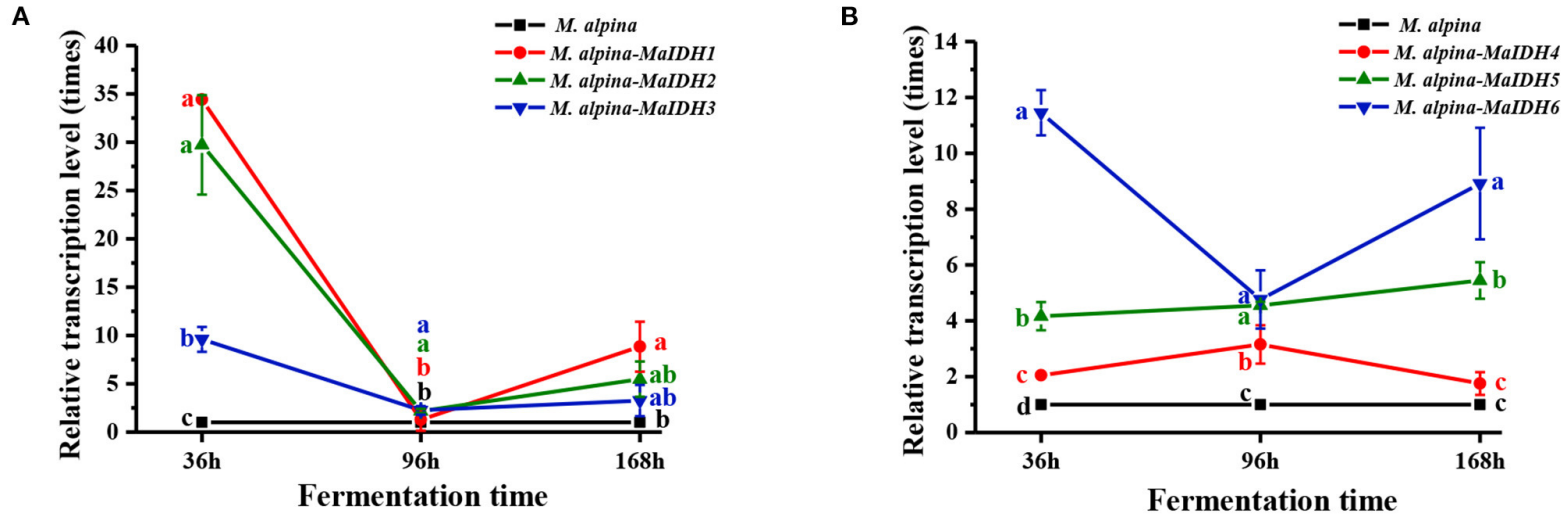
Transcriptional levels of various MaIDHs recombinant strains of *M. alpina* at different time points (36, 96, and 168 h). **(A)** Transcriptional levels of NAD^+^-MaIDHs, **(B)** transcriptional levels of NADP^+^-MaIDHs.

The transcription levels of each MaIDH in the overexpressed recombinant strain was higher than that in the control group at 36 h, indicating that the MaIDH was successfully overexpressed in the recombinant strain (Figure 4). As an oxidoreductase with in *vivo* regulatory functions, IDH plays an important role in energy metabolism and has a significant impact on the life activities of organisms. Because the expression of genes in the cell is divided into two levels of transcription and translation, the time and location of transcription and translation of eukaryotic genes are different. The formation of active proteins in the post-transcriptional translation process requires further processing and modification. Sometimes, when the protein level reaches its peak, its mRNA is degraded, so the transcription level of the gene is not entirely consistent with the expression level of the protein. Levitan et al. (35) found no change in the transcriptional level of malic enzyme after nitrogen limitation, but its protein expression level was significantly increased. Therefore, the transcript level results can preliminarily determine whether a gene plays a role, but further research is needed to understand its impact on the biological phenotype.

**Figure 5:**
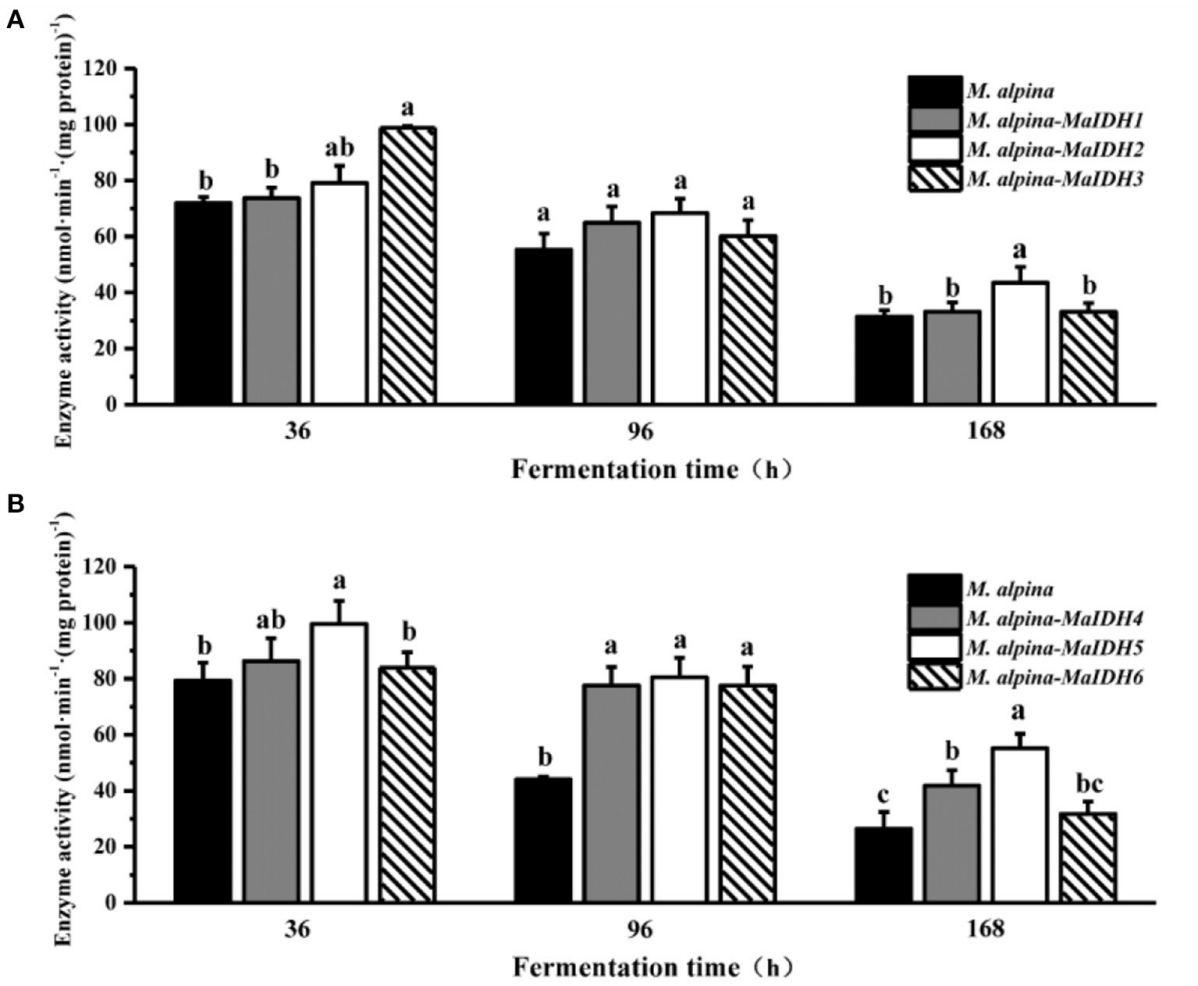
Enzyme activity of isocitrate dehydrogenases from *M. alpina* (MaIDHs) expressed by recombinant strains at different time points (36, 96, and 168 h). **(A)** Changes in enzyme activity of NAD^+^-MaIDHs, **(B)** changes in enzyme activity of NADP^+^-MaIDHs. Different letters indicate significant differences, *p* < 0.05.

The effects of overexpressing MaIDH in the recombinant strain of *M. alpina* were further investigated at the protein level by measuring the enzymatic activity of MaIDH in the recombinant strain (Figure 5). The enzyme activity of MaIDH in all recombinant strains gradually decreased over prolonged fermentation, but was still higher than that of the control group. During the fermentation process, the enzyme activity of the NAD^+^-MaIDH3 recombinant strain decreased from 98.8 nmol·min^−1^·(mg protein)^−1^ at 36 h to 60.1 nmol·min^−1^·(mg protein)^−1^ at 96 h, a 39.2% reduction. However, the changes in the enzymatic activity of NAD^+^-MaIDH1 and NAD^+^-MaIDH2 recombinant strains were not significant. This may be due to the fact that NAD^+^-MaIDH3 is more dependent on AMP concentration than NAD^+^-MaIDH1 and NAD^+^-MaIDH2; thus, its activity is more obviously decreased with nitrogen exhaustion. Previous studies have shown that different IDHs have different degrees of dependence on AMP. The Ratledge team conducted a preliminary investigation of mitochondrial NAD^+^-IDH in oil-producing and non-oil-producing yeast (23). The results showed that the activity of mitochondrial NAD^+−^IDH in oleaginous yeast was dependent on AMP, but that in non-oil producing yeast was not dependent on AMP. Furthermore, our previous study also indicated that the activity of the mitochondrial NAD^+^-IDH in the oleaginous filamentous fungus *Mucor circinelloides* is only dependent on AMP at low isocitrate concentrations, and its activity is not fully regulated by AMP (24). Our results showed that the enzyme activities of NADP^+^-MaIDH4/5/6 were obviously higher than those of the control strain, especially at 96 h. As *M. alpina* enters the lipid accumulation phase, it requires an intracellular supply of large amounts of NADPH for lipid synthesis, and the increased activity of NADP^+^-MaIDH is beneficial to lipid accumulation.

### Growth and Lipid Accumulation in the MaIDH Homologous Overexpressing Strain

**Figure 6:**
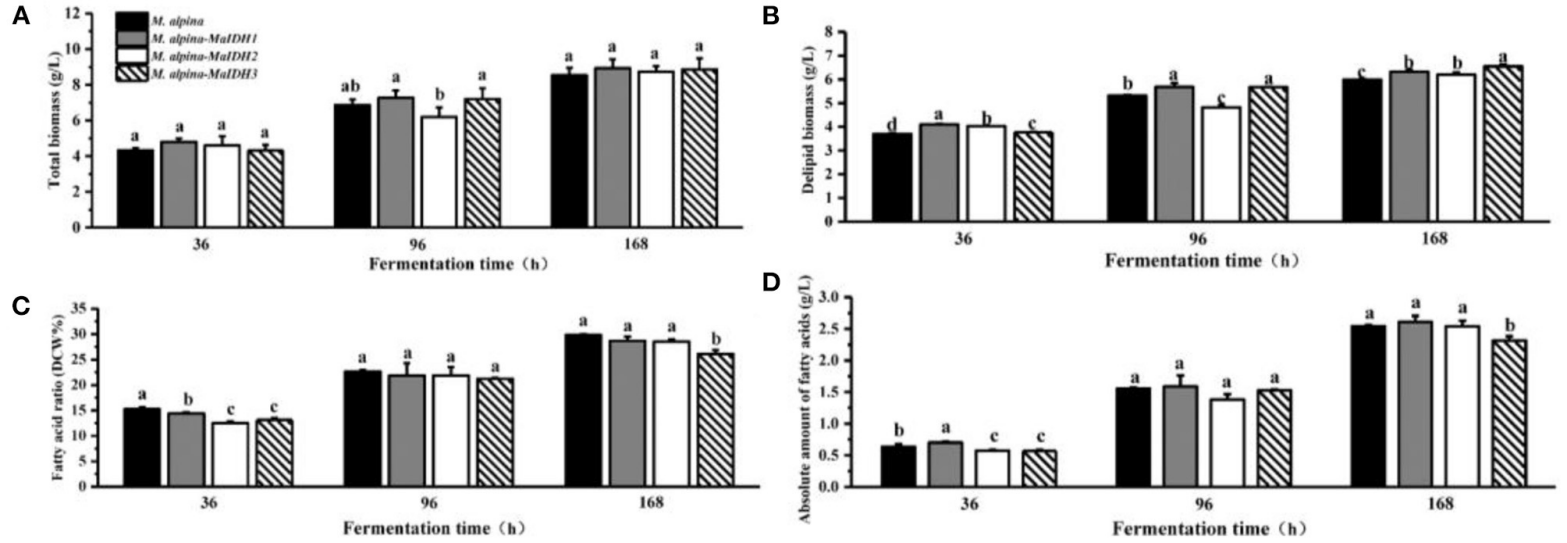
Biomass and lipid accumulation of recombinant strains overexpressing NAD^+^-MaIDH at different time points (36, 96, and 168 h). Different letters indicate significant differences, *p* < 0.05. **(A)** Total biomass, **(B)** delipid biomass, **(C)** fatty acid ratio, and **(D)** absolute amount of fatty acids.

**Figure 7:**
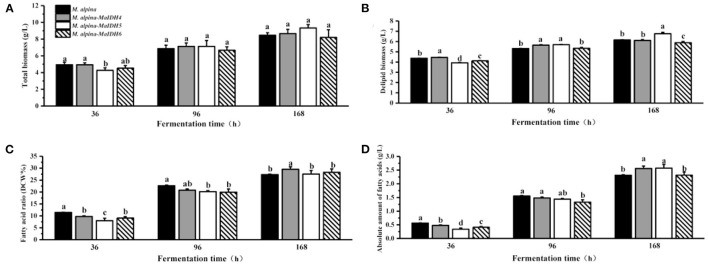
Biomass and lipid accumulation of recombinant strains overexpressing NADP^+^-MaIDH at different time points (36, 96, and 168 h). Different letters indicate significant differences, *p* < 0.05. **(A)** Total biomass, **(B)** delipid biomass, **(C)** fatty acid ratio, and **(D)** absolute amount of fatty acids.

To further investigate the effects of various isozymes of MaIDH on the growth and lipid accumulation in the recombinant strains, the biomass and fatty acid content at different time points in the fermentation process were determined (Figures 6, 7). As a result, it was found that the total biomass of each MaIDH homologous overexpressing strain during the fermentation did not change significantly compared with the control group and the values ranged from 8.5 to 9.3 g/L at the end of 168 h fermentation (Figures 6A, 7A).

According to the data on the fatty acid content of each recombinant strain, it can be seen that the recombinant strains of MaIDH have different effects on lipid accumulation. As shown in Figure 6C, the homologous overexpression of NAD^+^-MaIDH3 resulted in a significant decrease in fatty acid content in the recombinant strain, compared to the control group, which had decreased by 12.5% at 168 h. In contrast, overexpression of NAD^+^-MaIDH1 and NAD^+^-MaIDH2 had no effect on the fatty acid content. The results indicated that NAD^+^-MaIDH3 may play a major role in the three NAD^+^-MaIDH isoenzymes, a phenomenon that is also present in other proteins with isoenzymes and is a widespread self-protection and regulation mechanism in organisms (36). In addition, Yang et al. heterologously expressed NAD^+^-IDH from the oil-producing microorganism *Rhodosporidium toruloides* in *Saccharomyces cerevisiae* knocked out of IDH, and found that the fatty acid content of the recombinant strain was higher than that of the wild-type strain; which also indicated that NAD^+^-IDH can indeed affect lipid synthesis (37).

As shown in Figure 7C, among the recombinant strains overexpressing NADP^+^-MaIDH4/5/6, only NADP^+^-MaIDH4 enhanced the total fatty acid content at the end of fermentation for 168 h, which increased by 8.2%. Combined with the transcriptome data of *M. alpina* (33), it was found that the transcription level of MaIDH4 was upregulated after the nitrogen source was depleted, and its FPKM value was maintained at a high level throughout the fermentation process. Notably, overexpression of NADP^+^-MaIDH4 has a more pronounced effect on lipid accumulation than NADP^+^-MaIDH5/6, which may be related to its high transcription level. NADP^+^-IDH can provide the reducing power NADPH for fatty acid biosynthesis (38). Thus, our study indicated that NADP^+^-MaIDH4 may influence lipid synthesis by affecting intracellular NADPH content. In addition, the fatty acid composition in the recombinant strains was analyzed, and the results showed that overexpressed NADP^+^-IDH slightly decreased the ARA content in total fatty acids compared with the control strain (Table 2).

**Table 2:** Fatty acid composition (%, w/w of total fatty acids) of the recombinant strains during the fermentation.

**Strains**	**Fatty acid composition (%, w/w of total fatty acids)**							
**(at different time)**	**C 16:0**	**C 18:0**	**C 18:1**	**C 18:2**	**C 18:3**	**C 20:3**	**C 20:4**	**Others**
*M. alpina* (36 h)	14.7 ± 0.3	12.9 ± 0.1	11.7 ± 0.4	5.3 ± 0.1	5.6 ± 0.1	4.3 ± 0.1	38.6 ± 0.3	6.8 ± 0.1
*M. alpina* (96 h)	14.1 ± 0.3	12.2 ± 0.2	11.2 ± 0.5	9.7 ± 0.4	5.1 ± 0.1	3.3 ± 0.1	37.5 ± 1.1	6.9 ± 0.2
*M. alpina* (168 h)	15.9 ± 1.6	14.0 ± 1.5	13.5 ± 1.2	13.6 ± 2.1	4.8 ± 0.5	2.9 ± 0.2	28.8 ± 1.7	6.5 ± 0.1
*M. alpina-MaIDH1* (36 h)	14.6 ± 0.1	12.3 ± 0.2	14.0 ± 0.1	5.6 ± 0.1	5.3 ± 0.1	4.5 ± 0.1	37.5 ± 0.2	6.3 ± 0.1
*M. alpina-MaIDH1* (96 h)	15.1 ± 0.1	12.7 ± 0.5	14.0 ± 0.1	9.9 ± 0.4	5.0 ± 0.1	3.4 ± 0.1	33.4 ± 0.1	6.5 ± 0.2
*M. alpina-MaIDH1* (168 h)	15.3 ± 0.3	14.3 ± 0.5	15.4 ± 1.1	13.5 ± 0.6	4.6 ± 0.1	3.0 ± 0.1	27.5 ± 1.2	6.4 ± 0.1
*M. alpina-MaIDH2* (36 h)	14.4 ± 0.1	11.5 ± 0.6	13.5 ± 0.1	6.3 ± 0.3	5.2 ± 0.1	4.5 ± 0.2	38.0 ± 0.4	6.6 ± 0.1
*M. alpina-MaIDH2* (96 h)	15.1 ± 0.3	11.6 ± 0.3	13.6 ± 0.3	11.5 ± 0.7	4.4 ± 0.1	3.1 ± 0.1	33.7 ± 0.6	7.0 ± 0.2
*M. alpina-MaIDH2* (168 h)	15.5 ± 0.3	13.1 ± 0.3	14.9 ± 0.1	14.0 ± 0.3	4.1 ± 0.1	2.8 ± 0.1	28.9 ± 0.4	6.7 ± 0.1
*M. alpina-MaIDH3* (36 h)	14.4 ± 0.3	12.6 ± 0.4	12.1 ± 0.3	5.7 ± 0.2	5.4 ± 0.1	4.6 ± 0.1	39.0 ± 0.8	6.1 ± 0.1
*M. alpina-MaIDH3* (96 h)	14.6 ± 0.2	12.2 ± 0.4	12.1 ± 0.1	10.0 ± 0.2	5.2 ± 0.1	3.4 ± 0.1	35.9 ± 0.2	6.7 ± 0.1
*M. alpina-MaIDH3* (168 h)	15.1 ± 0.1	13.6 ± 0.4	14.0 ± 1.1	13.8 ± 0.9	4.6 ± 0.1	3.0 ± 0.1	29.4 ± 0.7	6.6 ± 0.2
*M. alpina-MaIDH4* (36h)	16.5 ± 0.4	14.0 ± 0.5	16.9 ± 0.4	6.4 ± 0.2	5.3 ± 0.2	3.9 ± 0.1	29.7 ± 1.0	7.1 ± 0.1
*M. alpina-MaIDH4* (96 h)	14.8 ± 0.4	11.5 ± 0.1	12.7 ± 0.5	12.9 ± 0.7	4.9 ± 0.1	2.8 ± 0.1	33.4 ± 1.3	7.2 ± 0.1
*M. alpina-MaIDH4* (168 h)	15.2 ± 1.1	14.0 ± 0.8	14.8 ± 0.1	14.2 ± 0.3	4.9 ± 0.2	2.7 ± 0.1	27.6 ± 1.6	6.7 ± 0.2
*M. alpina-MaIDH5* (36 h)	19.8 ± 0.6	16.0 ± 0.3	20.0 ± 0.4	4.7 ± 0.1	5.8 ± 0.3	4.9 ± 0.1	22.3 ± 0.9	6.6 ± 0.1
*M. alpina-MaIDH5* (96 h)	17.2 ± 0.3	11.6 ± 0.1	16.4 ± 0.2	11.6 ± 0.2	5.1 ± 0.1	3.4 ± 0.1	28.5 ± 0.4	6.3 ± 0.1
*M. alpina-MaIDH5* (168 h)	16.2 ± 0.2	13.3 ± 0.3	17.3 ± 0.2	13.8 ± 0.2	4.9 ± 0.2	3.1 ± 0.1	25.3 ± 0.1	6.1 ± 0.3
*M. alpina-MaIDH6* (36 h)	17.9 ± 0.1	15.3 ± 0.1	19.5 ± 0.2	5.0 ± 0.1	5.9 ± 0.2	4.9 ± 0.1	26.1 ± 0.2	5.4 ± 0.1
*M. alpina-MaIDH6* (96 h)	15.7 ± 0.9	14.9 ± 0.4	24.5 ± 1.0	7.5 ± 0.5	5.1 ± 0.4	3.3 ± 0.1	23.6 ± 1.5	5.4 ± 0.1
*M. alpina-MaIDH6* (168 h)	16.1 ± 0.9	15.1 ± 0.1	19.0 ± 3.6	10.9 ± 0.6	5.2 ± 0.3	3.0 ± 0.1	25.2 ± 1.4	5.5 ± 0.2

### Analysis of Intracellular Metabolites in the MaIDH-Overexpressing Strain

As MaIDH is not directly involved in fatty acid synthesis (e.g., fatty acid synthase, fatty acid desaturase), its regulation of lipid synthesis is carried out by intracellular citric acid content or the reducing power of NADPH. Therefore, the effect of each MaIDH isoenzyme on intracellular lipid metabolism can be further explored by assaying the substrates and products associated with MaIDH in the recombinant strain.

Citric acid is a key substrate that provides acetyl-CoA for fatty acid biosynthesis, and other metabolites from the TCA cycle also play critical roles in the lipid accumulation of filamentous fungi (24). In this study, the main metabolites from the TCA cycle in the recombinant strain overexpressing NAD^+^-MaIDH3 were analyzed at 168 h of fermentation. It can be seen from Table 3 that the citric acid content was increased, while isocitric acid, succinic acid, fumaric acid, malic acid, and pyruvic acid content in the recombinant strain was decreased compared to that in the control strain. These observations indicate that the overexpression of the NAD^+^-MaIDH3 strain resulted neither in greater carbon flux to the TCA cycle nor fatty acid biosynthesis. This is consistent with the result that overexpression of the NAD^+^-MaIDH3 strain had a low content of fatty acid accumulation.

**Table 3:** Changes in related metabolite of NAD^+^-MaIDH recombinant strains.

**Metabolite**	**Relative amount**
	**(*M. alpina*-*MaIDH3*/*M. alpina*)**
Citric acid	1.16 ± 0.04
Isocitric acid	0.90 ± 0.07
α-ketoglutarate	1.00 ± 0.05
Succinic acid	0.64 ± 0.08
Fumaric acid	0.86 ± 0.09
Malic acid	0.93 ± 0.03
Pyruvic acid	0.61 ± 0.04

With there being eight condensations reactions needed to produce a C18-fatty acyl-CoA, then 16 mol NADPH are needed. The source of this NADPH is then one of the major metabolic challenges in elucidating lipid biosynthesis in oleaginous microorganisms (39). According to the measurements of intracellular NADPH reducing power, the overexpression of NADP^+^-MaIDH4 in *M. alpina* could effectively increase intracellular NADPH content. As shown in Table 4, the NADPH content in the NADP^+^-MaIDH4 overexpressing strain increased from 36.5 to 50.3% compared to the control strain. The results showed that NADP^+^-MaIDH4 could effectively regulate intracellular NADPH content and increase lipid accumulation. Similar results were reported in previous studies. The study of Hao et al. found that overexpression of malic enzyme had a significant effect on NADPH production and lipid biosynthesis in *M. alpina* (29). Hao et al. also studied the source of NADPH in *M. alpina* and the results indicated that overexpression of the enzymes from pentose phosphate pathway could enhance NADPH contributor and increases fatty acid production (31).

**Table 4:** NADPH production in *M. alpina-MaIDH4* recombinant strains.

**Strains**	**NADPH/(NADPH+NADP^**+**^)**
	**(%)**
*M. alpina*	36.5 ± 6.2
*M. alpina-MaIDH4*	50.3 ± 3.9

## Conclusion

In this study, we constructed isoenzyme-overexpressing strains of MaIDH to explore their roles in lipid synthesis in *M. alpina*. Overexpressing NAD^+^-MaIDH3 reduced total lipid content due to its greater carbon flux with the tricarboxylic acid cycle and less carbon flux with fatty acid biosynthesis. While NADP^+^-MaIDH4 enhanced the total fatty acid content may be due to the great reducing power NADPH is produced in the overexpression strain. Our results provide theoretical support for the analysis of the lipid synthesis mechanism in *M. alpina*.

## Data Availability Statement

The original contributions presented in the study are included in the article/Supplementary Material, further inquiries can be directed to the corresponding author/s.

## Author Contributions

XT and XS: carried out the experiments and drafted the manuscript. XT, XS, and XW: analyzed the data and helped to draft the manuscript. HZ, YC, JZ, HC, and WC: conceived and designed the study and revised the manuscript. All authors contributed to the article and approved the submitted version.

## Funding

This research was supported by the National Natural Science Foundation of China (31901659 and 31722041), the project funded by China Postdoctoral Science Foundation (2017M611701), the Postdoctoral Science Foundation of Jiangsu Province (1701061C), the National First-class Discipline Program of Food Science and Technology (JUFSTR20180102), and the Jiangsu Province Collaborative Innovation Center for Food Safety and Quality Control.

## Conflict of Interest

The authors declare that the research was conducted in the absence of any commercial or financial relationships that could be construed as a potential conflict of interest.

## Publisher's Note

All claims expressed in this article are solely those of the authors and do not necessarily represent those of their affiliated organizations, or those of the publisher, the editors and the reviewers. Any product that may be evaluated in this article, or claim that may be made by its manufacturer, is not guaranteed or endorsed by the publisher.
