# Supplementary material for: Characterization of NAD+/NADP+-Specific Isocitrate Dehydrogenases From Oleaginous Fungus Mortierella alpina Involved in Lipid Accumulation
Source: Front Nutr. 2021 Oct 21;8:746342. doi: 10.3389/fnut.2021.746342 (PMC8566678; doi:10.3389/fnut.2021.746342)
Supplement: Supplementary file 1 [file Data_Sheet_1.DOCX]

**Table S1 Primers sequence for target genes**

| **Primer name** | **Primer sequence（5’-3’）** | **application** |
| --- | --- | --- |
| *MaIDH1*-F | CCCAAGCTTCAATGGGTCCTCGAGCCATGAGC | Obtain the target gene *MaIDH1* |
| *MaIDH1*-R | CAACCGAGCTCTTACAGGTTAGAGATGACGGCAAAC |  |
| *MaIDH2*-F | CCCAAGCTTCAATGGGTCCCCGTGCCATGA | Obtain the target gene *MaIDH2* |
| *MaIDH2*-R | CCGCTCGAGTTACAGGTTGGAGATGACAGCAA |  |
| *MaIDH3*-F | CCCAAGCTTCAATGTTTGCCACCCAGCGT | Obtain the target gene *MaIDH3* |
| *MaIDH3*-R | CCGCTCGAGTTACAGCTCGCTGATGATAGCGT |  |
| *MaIDH4*-F | CCCAAGCTTCAATGCTTGCCAACAAAATCAACGG | Obtain the target gene *MaIDH4* |
| *MaIDH4*-R | TCCCCCGGGTTAAACGGTGCGCTTCTTCTGC |  |
| *MaIDH5*-F | CCCAAGCTTCAATGAAAGACTCGAAGGATGCG | Obtain the target gene *MaIDH5* |
| *MaIDH5*-R | TCCCCCGGGTTAGACGCCACGGACCTT |  |
| *MaIDH6*-F | CCCAAGCTTCAATGACTCGCATCATTTGGGAC | Obtain the target gene *MaIDH6* |
| *MaIDH6*-R | TCCCCCGGGTCACAAGGCCGACTTGAGC |  |

The underlined part is the enzyme cleavage site.

**Table S2 Primer sequence for RT-qPCR**

| **Primer name** | **Primer sequence（5’-3’）** | **application** |
| --- | --- | --- |
| RT-*MaIDH1*-F | CAGTTCAGGCATCAAGTT | Obtain the target gene *MaIDH1* |
| RT-*MaIDH1*-R | TAGGCATGACCATGACAT |  |
| RT-*MaIDH2*-F | AACAAGGTTGGTCTCAAG | Obtain the target gene *MaIDH2* |
| RT-*MaIDH2*-R | AACAAGGGAAGCATACAT |  |
| RT-*MaIDH3*-F | GTCCTTATCCGTGAGAAC | Obtain the target gene *MaIDH3* |
| RT-*MaIDH3*-R | TCAGAAGCATCCTTAGTG |  |
| RT-*MaIDH4*-F | CCTATGTCGATCTTGATAT | Obtain the target gene *MaIDH4* |
| RT-*MaIDH4*-R | ACTTCTTAATTGCCTCAG |  |
| RT-*MaIDH5*-F | CAAGAACACTATCCTGAAGAAG | Obtain the target gene *MaIDH5* |
| RT-*MaIDH5*-R | AATCAATCGGTGCTCGTA |  |
| RT-*MaIDH6*-F | GTGATTACAAGCAACAGT | Obtain the target gene *MaIDH6* |
| RT-*MaIDH6*-R | CCGAGGACTTTAACATCT |  |
| RT-18S *rDNA* F | CGTACTACCGATTGAATGGCTTAG | 18S rDNA reference gene |
| RT-18S *rDNA* R | CCTACGGAAACCTTGTTACGACT |  |
